# Intrageneric differences in the effects of acute temperature exposure on competitive behaviour of damselfishes

**DOI:** 10.7717/peerj.7320

**Published:** 2019-07-17

**Authors:** Donald T. Warren, Mark I. McCormick

**Affiliations:** College of Science and Engineering, James Cook University, Townsville, Queensland, Australia

**Keywords:** Climate change, Competition, Temperature, Thermal plasticity, Coral reef fish

## Abstract

Projected increases in global temperatures brought on by climate change threaten to disrupt many biological and ecological processes. Tropical ectotherms, like many fishes, can be particularly susceptible to temperature change as they occupy environments with narrow thermal fluctuations. While climate change models predict temperatures to increase over decades, thermal fluctuations are already experienced on a seasonal scale, which may affect the ability to capture and defend resources across a thermal gradient. For coral reef fish, losers of competitive interactions are often more vulnerable to predation, and this pressure is strongest just after settlement. Competitive interactions may determine future success for coral reef fishes, and understanding how temperature experienced during settlement can influence such interactions will give insight to community dynamics in a future warmer world. We tested the effect of increased temperatures on intraspecific competitive interactions of two sympatric species of reef damselfish, the blue damselfish *Pomacentrus nagasakiensis*, and the whitetail damselfish *Pomacentrus chrysurus*. Juvenile fishes were exposed to one of four temperature treatments, ranging from 26–32 °C, for seven days then placed into competitive arenas where aggressive interactions were recorded between sized matched individuals within each species. While there was no apparent effect of temperature treatment on aggressive behaviour for *P. chrysurus,* we observed up to a four-fold increase in aggression scores for *P. nagasakiensis* with increasing temperature. Results suggest that temperature experienced as juveniles can impact aggressive behaviour; however, species-specific thermal tolerances led to behavioural affects that differ among closely related species. Differential thermal tolerance among species may cause restructuring of the interaction network that underlies the structure of reef assemblages.

## Introduction

Temperature is an important abiotic factor that can have profound effects on behaviour, physiology, and life-history traits (Schulte, Healy & Fangue, 2011). For many species, temperature dictates basic daily activities such as locomotion (Rome, 2007), aggression (Pruitt, Demes & Dittrich-Reed, 2011), and boldness (Biro, Beckmann & Stamps, 2010). Currently, climate change models predict sea surface temperature will rise as much as +3 °C by 2100 (Collins et al., 2013), impacting the ecology of many species (Walther et al., 2002). Yet, changes in temperature already occur temporally on a daily and seasonal scale or spatially such as across latitude or with water depth. Even within tropical ecosystems, which are considered relatively thermally stable, coral reefs can experience seasonal thermal variation of 8 °C (McCabe et al., 2010). This effect of temperature change can be more pronounced in ectotherms as their metabolism tends to increase nearly exponentially with rising temperature (Clarke & Johnston, 1999). Studying how reef species respond to temperature change can be helpful for determining the impact future climate change will have.

While climate change models predict temperature change to occur over several decades, the effect of warmer temperatures on reef fish can be observed today on a seasonal scale. Many benthic marine species have a planktonic larval phase that coincides with the lunar cycle, where juveniles recruit back to the reef to join the adult population (Leis & McCormick, 2002). This oscillation with moon patterns results in pulses of newly settling juveniles over the reproduction season. Cohorts of juveniles that settle at the beginning of the season will experience cooler temperatures, have less competition for shelter sites, and will have a head start on growth. Comparatively, cohorts that settle later in the season will experience warmer temperatures, must compete for shelter sites, and will be behind on growth. As temperatures continue to rise in line with climate projections, temperatures normally experienced during mid-late summer may occur earlier in the season. Determining the effects of temperature experienced by newly recruiting fishes on individual performance will give insight to how increases in future average temperatures may affect coral reef dynamics.

The effects of environmental temperature on individual performance will be particularly important at life-history bottlenecks (Allan et al., 2015). When fish settle to a juvenile population on a reef at the end of the larval phase over half of juveniles are consumed by predation in the first two days (Almany & Webster, 2006). Juveniles that acquire and defend a shelter have a greater chance of survival. In general, relatively dominant individuals can secure shelter sites that provide better refuge from predators and access to food sources compared to their subordinate counterparts (McCormick, 2009; McCormick, 2012). This creates a selective bottleneck on juveniles where aggression and competitive dominance can generate an advantage. The temperature experienced during this phase has previously been shown to influence aggressive interactions in two damselfish, *Pomacentrus amboinensis* and *Pomacentrus moluccensis* (Warren et al., 2016). This study suggested a species-specific response to elevated temperature as *P. amboinensis* increased aggressive behaviour with temperature while aggression scores in *P. moluccensis* decreased (Warren et al., 2016). Determining how temperature may affect these aggressive interactions in fish will aid our understanding of how reef community dynamics may be affected over seasonal temperature fluctuations and also will be informative in a climate change context.

The present study explores how temperature experienced during recruitment can impact intraspecific competitive interactions in two common congeneric damselfish, the whitetail damselfish *Pomacentrus chrysurus* and the blue damselfish *Pomacentrus nagasakiensis*. Damselfish represent a useful model as they have similar life-history characteristics to many coral reef fishes, but recruit in sufficiently large numbers to enable manipulative experiments. Larvae settle to specific habitats after a dispersive larval phase and aggressively defend these areas against similar sized fishes (e.g., McCormick & Weaver, 2012). We predicted that aggressive interactions would be lowest at ambient temperatures and increase as treatment temperature moved further from their ambient condition. Futhermore, it is likely that the species have different thermal tolerances, therefore we predicted the magnitude of change in aggression with temperature may be differ.

## Methods

### Study species, collection, and holding facilities

The study species were the whitetail damselfish, *P. chrysurus*, and the blue damselfish, *P. nagasakiensis.* These species are found throughout the Great Barrier Reef (Allen, 1991) and have been the focus of multiple ecological and behavioural experiments (e.g., Chivers et al., 2014; Chivers et al., 2019; Ferrari et al., 2011). Data on the species’ occurrence suggests similar temperature tolerances with both fish ranging from the Tropic of Capricorn across the equator (*P. nagasakaniensis*, 25°S to 10°N; *P. chrysurus*, 23°S to 30°N) (Allen, 1991). Newly metamorphosed juveniles (3–4 weeks old; standard length, $\overline{\mathrm{x}}$ ± SD; *P. chrysurus* 13.26 ± 0.78 mm; *P. nagasakiensis* 15.46 ± 0.97 mm) were collected using light traps (Meekan et al., 2001) at Lizard Island on the northern Great Barrier Reef (14.68°S, 145.47°E), Australia during November 2015. Light traps allow researchers to collect individuals of the same cohort in a single night. This means specimens are obtained prior to the high and selective mortality that occurs at the settlement transition (e.g., Almany & Webster, 2006; Holmes & McCormick, 2010), which represents an important life-history bottleneck. This is especially useful when studying competitive behaviour as previous interactions can produce a “winner effect” and influence the outcome of subsequent interactions (Poulos & McCormick, 2014).

**Figure 1:**
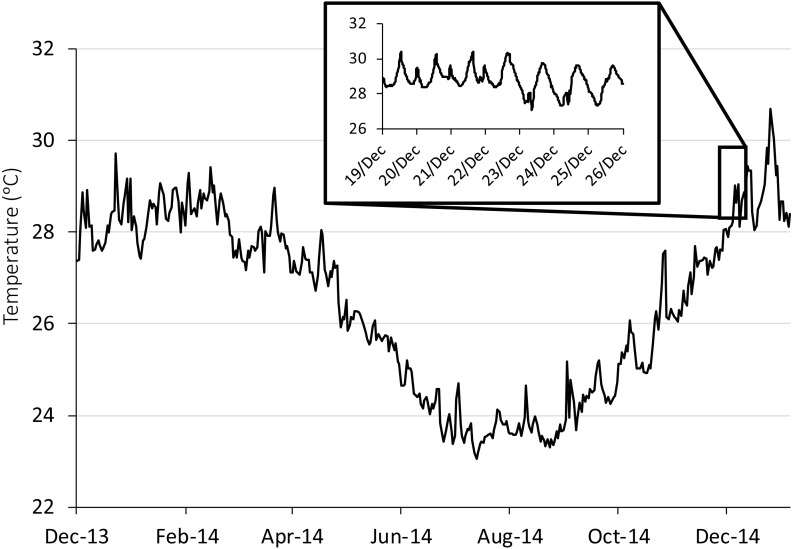
Seasonal and diurnal temperature fluctuation at Lizard Island, northern Great Barrier Reef. Seasonal fluctuation (23–30.6 °C) from daily means recorded by long-term temperature loggers (Tinytag, Gemini Dataloggers UK Ltd) positioned on a shallow (2 m) backreef from December 2013 to December 2014. Inset shows the daily fluctuation (27.1–30.4 °C) for the collection season, recorded every 20 min.

Study individuals were transported to the laboratory in 60 L containers of aerated seawater. Upon arrival, researchers carefully used hand nets to randomly sort fish into 16 32 L holding tanks (*n* = 25) for a total of 200 individuals per species. The 16 tanks were then randomly assigned to one of 4 temperature treatments: 26, 28, 30, and 32 °C. Ambient temperatures (control) at the time of the experiment averaged 28 °C. The temperatures chosen for the experiment spanned the temperature fluctuation for the reproduction season (Oct–Jan). Temperature data was based on temperature loggers deployed around Lizard Island (Fig. 1) and verified as standard for the season against long-term loggers (AIMS, 2014). The 32 °C treatment represented potential maximum summer temperatures for 2100 (Collins et al., 2013). To reduce thermal stress, tank temperatures were changed 1.5 °C/day from ambient and held at treatment temperature for 7 days prior to testing (as per Gardiner, Munday & Nilsson, 2010). Holding temperatures and photoperiods fluctuated ±0.5 °C around the mean and 12:12 hr (07:00 –17:00), respectively. Fish were fed *Artemia* nauplii to *ad libitum* twice during daylight hours. All work reported herein was conducted under field permits and approvals from the James Cook University Animal Ethics Committee (approval A2080), the Great Barrier Reef Marine Park Authority (13/35909.1), and Queensland Fisheries (170251).

### Experimental design

The effect of temperature on each species was measured by conducting intraspecific contests within temperature treatments. No trials occurred between fish from the same holding tank to control for the possibility of a pre-established hierarchy, and fish were used only once in the study to maintain novelty of the design and prevent any winner effects (Poulos & McCormick, 2014). In total, there were eight treatment combinations: 4 temperatures ×2 species. Previous work investigating changes in aggressive behaviour with temperature obtained significant findings with 10 replicates (Warren et al., 2016). The current study aimed to double this to increase statistical power. Replicates ranged from 17–20 pairs per treatment due to mortality in captivity and adverse events during trials (e.g., fish escape).

Size differences between competitors can influence the outcome of competitive interactions (McCormick, 2009; McCormick & Weaver, 2012). To control for this size effect, fish were measured and pairs were matched within 10% of their standard length (Warren et al., 2016). One day prior to experimental trials, fish were randomly collected from the appropriate holding tanks using a jar (to minimize stress), placed in small plastic bags of aerated seawater and measured (standard length) with callipers. Fish were then kept overnight in 1L aquaria of aerated seawater of the appropriate temperature to de-stress prior to their use in the competition trials. This process enabled the rapid collection of size-matched individuals prior to each trial. Fish were fasted for 12–24 h prior to competitive trials.

### Competitive interaction trials

**Figure 2:**
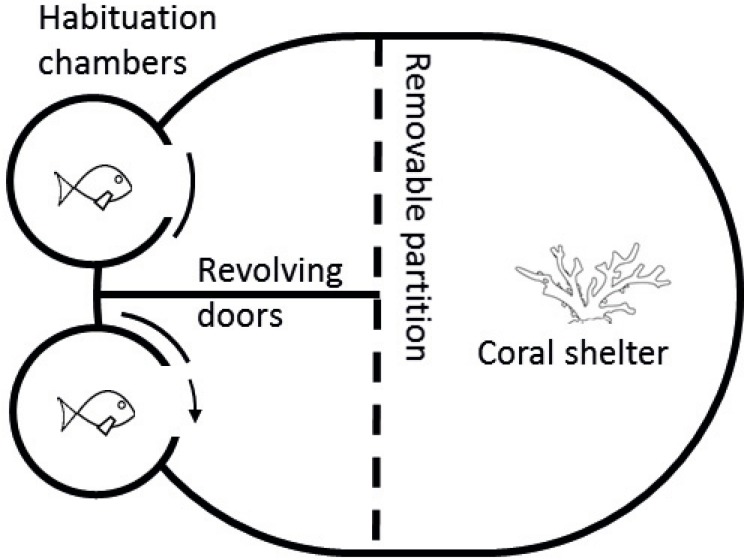
Diagram of competition arena. Size-matched pairs were placed into habituation chambers at the beginning of a trial for 5 min. Next, revolving doors were opened simultaneously, allowing fish to exit and explore their portion of the arena. Once both fish had emerged, video recording commenced from an above camera and the clear removable partition was lifted, exposing fish to the coral shelter and each other. A 5-min recording was taken starting from the first interaction. Videos were later analysed and three interactions were quantified: (i) displays, defined as a lateral fin flare towards the opponent; (ii) attacks, defined as a chase or biting of the opponent; and (iii) avoidances, swimming away from an opposing attack or display.

Competitive arenas and procedures were based on Chivers et al. (2017) and modified from Killen et al. (2014). Arenas consisted of an oval tank (37 × 30 cm) filled with seawater of the appropriate temperature to a depth of 10 cm with a fragment of live coral (*Pocillopora damicornis* ∼five cm^3^) in one half and two habituation chambers in the other half (Fig. 2). The coral provided shelter and served as a resource for competition. All trials were conducted during daylight hours (08:00–16:00). At the start of each trial, a size-matched pair of fish was transferred via water-filled jars to separate habituation chambers for 5 min. After the habituation period, revolving doors on both chambers were slowly opened simultaneously, allowing the two fish to emerge and explore their portion of the arena. Habituation chambers were separated by a solid fixed divider, preventing fish from seeing each other, thus making emergence an independent decision. A transparent and perforated partition allowed fish to view the coral on the other side but stopped either fish from reaching the shelter before the other. This partition prevented a priority effect of previous residency, a result known to influence outcomes of competitive interactions (Almany, 2003; Geange et al., 2016). Once both fish exited their chambers, the clear partition was carefully raised, exposing both fish to one another as well as allowing direct access to the coral fragment. Competitive interactions were video-recorded from the first encounter for 5 min and later analysed. Researchers were visually shielded from the fish by solid tank walls when opening habituation chambers and when removing the clear partition. Video recordings were started from outside the laboratory to further reduce the effect of observer disturbance on trials. Behavioural data was obtained from the video recordings, which were analysed blind of replicate and treatment. Three aggressive behaviours were quantified during video analysis: (i) displays, defined as a lateral fin flare towards the opponent; (ii) attacks, defined as a chase or biting of the opponent; and (iii) avoidances, swimming away from an opposing attack or display. A competitive trial generally consisted of multiple “face-offs”, where individuals would alternate between exploring the arena and approaching the other fish for an aggressive encounter. These encounters tended to begin with competitors displaying to each other by swimming side-by-side and head-to-tail in a circle. The display appears to be a non-physical attempt to convince its competitor of dominance status and coerce them into submission. If there was not an obvious dominant after the display, then the interaction escalated to chases, and then to bites. Once all three behaviours were quantified for both competitors, these variables were used to calculate an aggression score = attacks + displays—avoids (McCormick, 2009; Warren et al., 2016). The individual with the higher aggression score was deemed dominant and the contest winner for that pair. Aggression was used as a measure for competitive performance as it provides a good indicator of contest outcomes (McCormick, 2009; McCormick & Weaver, 2012).

### Statistical analysis

Aggression scores for each competitor were calculated, and the difference, taken as the winner score minus the loser score, was computed. Aggression score differences were analysed with a two-way ANOVA using species and temperature as fixed factors. Differences in size for each pair, calculated as winner fish size minus loser size, were normally distributed around the mean ($\overline{\mathrm{x}}=0.051$ mm, *SE* = 0.028) and did not differ from 0 (one-sample *t*-test, *t* = 1.72, *df* = 157, *p* = 0.08). A paired sample *t*-test using dominant and subordinate fish sizes was also used to explore whether there was a significant difference in sizes by dominance status. This too showed no significant difference (*t* = 1.83, *df* = 165, *p* = 0.068). However, when both analyses were split by species, there was a significant difference in the paired sample *t*-test between sizes of dominant and subordinate *P. nagasakiensis* (*t* = 2.44, *df* = 87, *p* = 0.017). To ensure that size matching was successful and the remaining difference in sizes did not decide contest outcomes, size differences in pairs were included as a covariate to aggression score differences. No effect of size was evident in the analysis and this term was subsequently dropped in the reported results. Aggression score differences can be driven by one competitor alone or confounded by a pair eliciting high numbers of aggressive behaviours. To explore this, separate two-way ANOVAs, one for each species, were undertaken with individual aggression score as dependent variable and competitor status (categorised as dominant or subordinate) and temperature as fixed factors.

The proportions of attacks, displays, and avoids elicited by each competitor were analysed using a two-way MANOVA to determine how temperature affected the dynamics of a contest. Temperature and competitor status were included as fixed factors and the proportion of interactions for each of the three behaviours as the dependant variables, with separate models generated for each species. Any significance found was further investigated with Tukey’s HSD post-hoc analyses. Bonferroni correction was used for the interpretation of hypothesis tests on individual variables for each species (Adjusted *α* = 0.017). This analysis examined how temperature affected the dynamics of a contest and also determined whether the aggression equation was an appropriate representation of aggressive behaviours during a competitive trial. Preliminary analyses tested the presence of a tank effect by including holding tank as a random factor. However, this did not change the results, so holding tank was excluded from subsequent analyses. Data were found to conform to the assumptions of normality and homogeneity using residual analyses.

## Results

**Figure 3:**
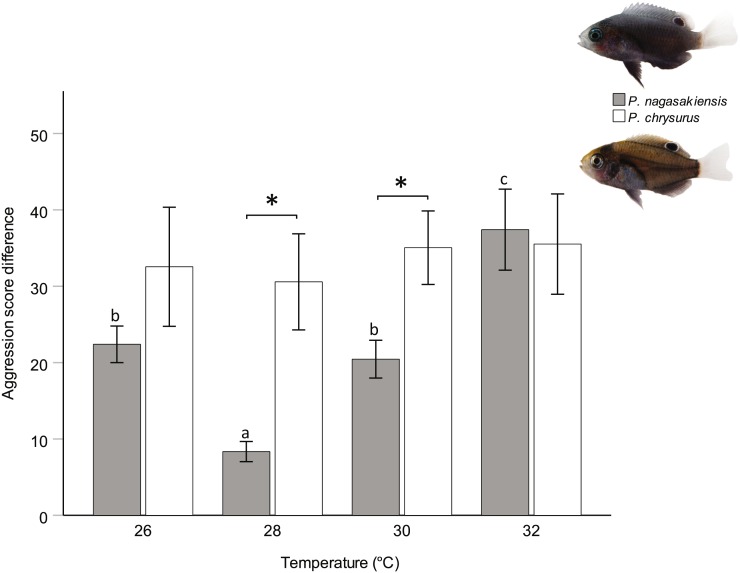
Influence of temperature on aggression scores between dominant and subordinate fish. Mean aggression score difference (±SE), taken as aggression score of dominant competitor minus the subordinate score, for intraspecific contests at each of four temperatures. Only *P. nagasakiensis* (solid) showed an effect of temperature with aggression lowest at ambient and increasing in either direction. Italicised letters above error bars represent Tukey’s HSD means comparisons. *P. chysurus* (open) was unaffected by temperature and aggression scores significantly differed from *P. nagasakiensis* at ambient and 30 °C (shown by *). *n* = 17–20 pairs. Photographic credits: MI McCormick.

**Figure 4:**
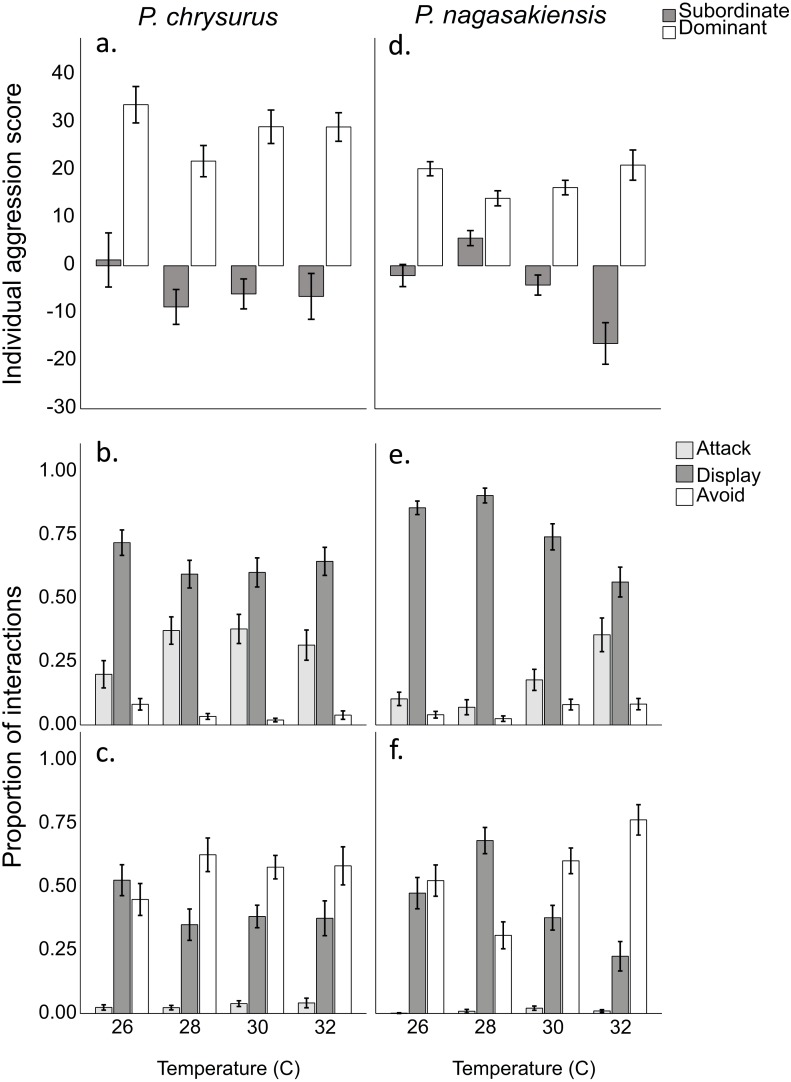
Aggression score and proportion of interaction behaviour by each competitor. (A) Mean aggression scores (±SE) of dominant (open) and subordinate (solid) competitors for intraspecific contests involving *P. chysurus* (A, B, C) or *P. nagasakiensis* (D, E, F). *P. nagasakiensis* showed a significant effect of temperature on aggression score with subordinate competitors more affected compared to dominants. (B) Mean proportion (±SE) of attacks (shaded), displays (solid), and avoids (open) elicited by the dominant competitor. (C) Mean proportions of behavioural interactions elicited by subordinate competitors. As temperature increased dominant *P. nagasakiensis* decreased displaying behaviour and increased proportions of attacks while subordinates decreased displays but increased number of avoids.

There were marked changes in species’ aggression score differences in response to temperature. Two-way ANOVA showed a significant effect of temperature (*F*_3,145_ = 3.80, *p* = 0.012) and species (*F*_1,145_ = 10.24, *p* = 0.002), but did not find a significant interaction between these two. The one-way ANOVA for *P. nagasakiensis*, revealed aggression scores were lowest at ambient (28 °C) and increased as temperature moved from ambient in either direction (*F*_3,73_ = 14.78, *p* < 0.001; Fig. 3). Conversely, aggression score differences for *P. chrysurus* were unaffected by temperature and were significantly greater than *P. nagasakiensis* at ambient and 30 °C (signified with * in Fig. 3). When aggression score differences were separated by competitor (i.e., dominant and subordinate scores), dominant *P. nagasakiensis* scores tended to increase with temperature while subordinate scores decreased. This change was more apparent in subordinate competitors as their aggression was significantly lowered by temperature (*F*_1,146_ = 12.45, *p* < 0.001; Fig. 4D). Temperature also affected the proportion of interactions elicited by each competitor during contest of *P. nagasakiensis* (*F*_6,290_ = 6.91, Wilk’s Λ = 0.76, *p* < 0.001). At elevated temperature, dominant *P. nagasakiensis* decreased displays *F*_1,73_ = 11.98, *p* < 0.001) and increased attacking behaviour (*F*_1,73_ = 8.69, *p* < 0.001; Fig. 4E). Concurrently, subordinate *P. nagasakiensis* decreased displays (*F*_1,73_ = 11.64, *p* < 0.001) and increased avoidance behaviour (*F*_1,73_ = 10.92, *p* < 0.001; Fig. 4F). For *P. chrysurus,* neither dominant nor subordinates showed a change in interaction proportions across temperatures (Figs. 4B and 4C, respectively).

## Discussion

Determining the effect of temperature on ecological processes is crucial for making realistic predictions about how communities may change in response to a warming ocean (Walther et al., 2002). While previous studies have looked at the effects of temperature on aggressive interactions in freshwater systems (De Staso & Rahel, 1994; Reese & Harvey, 2002; Taniguchi et al., 1998), few have focused on marine species (although see Pruitt et al., 2018). The current study found that aggression levels during competitive contests can increase with temperature experienced during settlement. Furthermore, the effect that temperature will have can be specific to each species. The effect of temperature on competition suggests that aggression may increase as summer averages increase with climate change. However, the species-specific response illustrates that differential thermal tolerance may lead to restructuring of reef-assemblages and dominance hierarchies within reef communities.

We found aggression scores differences for *P. nagasakiensis* were lowest when recruits were exposed to ambient temperature and increased when temperature changed in either direction. Increasing aggression with temperature is expected as activity level, boldness, and aggression have previously been shown to increase with temperature in a closely related damselfish (Biro, Beckmann & Stamps, 2010; McCormick & Meekan, 2010). This may be due to the increased metabolism that coincides with rising temperatures for ectotherms (Clarke & Johnston, 1999). When aggression score differences were separated into individual aggression score components (i.e., dominant and subordinate), we found that temperatures above ambient affected both competitors, though was greater for subordinates. The effect of temperature on both individuals shows aggression score differences were not driven by a single competitor. The greater effect on subordinates may be due to an increasing stress of losing at elevated temperatures. Losing a competitive contest can be stressful for an individual as subordinates will spend the duration avoiding aggressive displays and being denied access to shelter (Senar et al., 2000). This stress can manifest as elevated cortisol levels and metabolic rates (McCormick, 2016), which may make them less resilient to other stressors such as temperature change. However, aggression showed a similar increase at 2 °C below ambient (26 °C) as 2 °C above (30 °C). This change in aggressive performance contradicts trends predicted for physiological performance potentially indicating reducing temperature was more important to the behavioural response. It would be interesting whether this trend continues at 4 °C below ambient where aggression at 24 °C are similar to that found at 32 °C.

Temperature also impacted the dynamics of contests through altering the proportions of competitive behaviours elicited by each competitor. At ambient temperature, dominant competitors won an encounter by displaying most often, followed by attacking, and avoiding least often. Conversely, subordinates showed avoidance behaviour most often, then displays, and attacked least of all. This is different to previous research on competitive interactions in other species of damselfish showing dominants win contests by attacking most, then displaying (Killen et al., 2014; Warren et al., 2016). As water temperature increased, there was a shift in the behaviour of dominant *P. nagasakiensis* to exhibit less displays and more attacks, while subordinates increased avoids and displayed less. This suggests that higher temperatures can both increase overall aggression during contests as well as lead to fiercer competition. Aggressive interactions can already be costly in energy expenditure and increase risk of injury (Briffa & Sneddon, 2007). This transition to fiercer competition means individuals may be investing even more into contests, leaving fewer resources for other activities.

When comparing species, temperature had little effect on aggression score differences for *P. chrysurus* and was overall greater than *P. nagasakiensis* at 28 (ambient) and 30 °C*.* This shows a species-specific response to elevated temperature within congeneric species. Specificity by species to temperature has been shown in other work on competition in the damselfish *Pomacentrus amboinensis* and *P. moluccensis* (Warren et al., 2016). Here, there was opposing trends in aggression after short-term exposure to elevated temperature. Differences in aggression help demonstrate a species’ thermal performance across temperatures and may give insight to how species may fare with future warming. The increase of aggression with temperature for *P. nagasakiensis* suggests the thermal optimum for this species may be above current summer average with future warming potentially yielding better performance. Comparatively, the lack of change in aggression suggests *P. chrysurus* may have a broader thermal optimum with changes in temperature showing little effect on performance.

## Conclusions

Increasing temperature impacted aggressive contests and resulted in fiercer competition, however, the effect of temperature was specific to each species. Differences in thermal performance between species is most likely ubiquitous for fishes within trophic groups. Species that are dominant at current-day temperatures may be outcompeted at warmer temperatures, causing a shift in the dominance hierarchy within assemblages and potentially the distribution of species. Future studies could investigate the physiological mechanisms that underlie these changes in competition and how our observed changes in aggressive behaviour may cascade through the reef community. These studies should include competing species in interspecific trials, direct assessment of temperature on physiology, and longer-term exposure periods to temperature to give a more comprehensive picture of how climate change might be expected to influence the composition of coral reef fishes at particular geographic locations.

## Additional Information and Declarations

### Field Study Permissions

The following information was supplied relating to field study approvals (i.e., approving body and any reference numbers): The Directors of the Lizard Island Research Station gave permission to undertake the research. Work was undertaken under permits from the Great Barrier Reef Marine Park Authority (G13/35909.1), and Queensland Fisheries (170251).

### Data Availability

The following information was supplied regarding data availability: McCormick, M.; Warren, D. (2019): Data for: Warren DT, McCormick MI. Intrageneric differences in the effects of acute temperature exposure on competitive behaviour of damselfishes. James Cook University. (dataset). http://dx.doi.org/10.25903/5cad5e22a7f35.
